# Family Members’ Help-Seeking Behaviour for Their Relative Who Uses Substances: A Cross-Sectional National Study in Brazil

**DOI:** 10.3390/ijerph22060968

**Published:** 2025-06-19

**Authors:** Cassandra Borges Bortolon, Martha Canfield, Maria de Fatima Rato Padin, Jim Orford, Ronaldo Laranjeira

**Affiliations:** 1 Department of Psychiatry, Federal University of São Paulo, São Paolo 04021-001, Brazil; ratopadinfatima@gmail.com (M.d.F.R.P.);; 2 Acurarte, Psicologia, Ensino e Saude, Porto Alegre 90035-190, Brazil; 3 Department of Psychology, Glasgow Caledonian University, Glasgow G4 0BA, UK; martha.canfield@gcu.ac.uk; 4 School of Psychology, University of Birmingham, Birmingham B15 2TT, UK; j.f.orford@bham.ac.uk

**Keywords:** affected family members, substance use, Brazil, help seeking, cross-sectional study

## Abstract

The affected family members (AFM) of relatives with substance use problems (RSU) play an important role in supporting their relatives to enter substance use treatment. This study investigated the help-seeking behaviours for their relatives by AFM in Brazil, including the characteristics of those who sought help and the risk factors for delaying it. A secondary analysis from a national cross-sectional study of 3030 AFM was performed. Participants were recruited from a range of services focused on AFM across each of the five Brazilian regions (North, Northeast, Central-West, Southeast, South). While 92.7% sought help, 66.0% delayed for an average of 37.2 (SD 70.71) months. Help seeking was associated with higher socioeconomic status and being from the Southeastern region. Barriers included the relative refusing help (31.5%) and the belief that help was not needed (20.6%). Longer delays were associated with female AFM, residents in the Central-West region, non-parents, older RSU, alcohol use, and withdrawal coping strategies. The findings show disparities in help-seeking behaviour across socioeconomic groups, regions, and substance types, highlighting the need for better healthcare workforce distribution and targeted interventions to educate AFMs on the importance of engagement with healthcare services.

## 1. Introduction

Problematic substance use is a significant risk factor for a range of negative outcomes not only for individuals, but also for those who care for them. Research consistently demonstrates the adverse effects experienced by affected family members (AFM) of a relative with substance use problems (RSU). These effects include mood disorders and anxiety disorders, poor overall health status, frequent arguments and threats, financial strain, feelings of loneliness and fear, neglect, and domestic violence [1,2,3]. The data also suggest that the burden of caring for a relative is higher for AFM of RSU compared to those with other mental health disorders [4,5]. As a result, AFM experience higher healthcare utilisation, medical treatment costs, and productivity losses than family members not affected by an RSU [2,6,7]. While the actual prevalence of AFM remains unclear, studies conducted in Germany and Brazil estimate a prevalence of 9.5% (6.8 million) and 13% (28 million), respectively, in the population of these countries [8,9].

It is understood that the burden of caring for an RSU is universally experienced [1]. Nevertheless, it is also known that AFMs’ experiences are influenced by the cultural context, family structures, and societal perceptions of substance use [5]. Such factors are understood to shape how AFM learn to cope with and manage the stressors associated with the substance use problem [10]. Broadly, the research suggests that families who actively seek assistance and adopt new coping strategies experience a shift from feeling powerless to experiencing empowerment [10]. However, substance use is regarded as a highly stigmatised behaviour, which is a major barrier to help-seeking and behaviour change in AFM [11,12].

Considerable evidence points to the active role of AFM in supporting behaviour change in their relatives, including supporting them to enter substance use treatment [13,14]. A series of interventions for AFM of treatment-refusing individuals with substance use problems has been developed [15,16]; however, this has been restricted to high-income nations including Germany [17], Spain [18], and the USA [16,19,20]. Across Brazil and other low–middle-income countries, research to date has revealed little about the needs and difficulties that AFM face when seeking treatment support for their RSU. Similar to international trends [21], only a small minority (approximately 4.3%) of Brazilian individuals with substance use problems receive treatment [22]. Those who entered treatment often credited family members as the main reason for seeking professional help [23].

In Brazil, major gaps in availability and access to substance use treatment services exist, especially for marginalised people who use substances [24,25,26]. As a result, Brazilian AFM tend to seek out different systems of treatment for RSU (religious groups, mutual self-help groups, non-governmental organisations, and private and public health services) [27]. In addition, despite the national mental health and substance use treatment system in Brazil undergoing considerable changes over the past 20 years (towards a more patient/need-oriented system and accessible services within local communities), the country still lacks clear guidelines and regulations for the treatment of substance use [28]. This includes a lack of standardised frameworks to treat substance use, the absence of an official national committee overseeing substance use treatment services, and a lack of interventions and professional training aimed at working with AFM [28,29].

It is important to highlight the fact that the provision of services for individuals who use substances and their family members can vary considerably within the country regions. However, most studies on AFM tend to focus on the national level. This approach can limit our understanding of heterogeneities, especially when considering large countries. Brazil is an emblematic case. It has an area almost as extensive as Europe and more than 200 million inhabitants. Internal economic, social, and cultural differences are equally huge. For instance, the Gross Domestic Product (GDP) and Human Development Index (HDI) are significantly lower in the Northern regions of the country than in the Southern regions, with the Southeast being the region where the income distribution is most concentrated [30]. Thus, to understand the help-seeking behaviours of Brazilian AFM, it is necessary to consider possible regional differences.

The current study aimed to explore whether psycho-social adversities impact how Brazilian AFM seek help for their RSU. We conducted an exploratory analysis of a diverse and large sample of AFM considering factors such as type of relationship, geographic area, and the type of substance used by the relative. First, we explored the characteristics of those who sought help for their relative. We then explored risk factors for seeking help immediately after discovering the substance use problem and risk factors for delayed help seeking. Addressing these questions is vital to developing and adapting appropriate support and education programmes for AFM in Brazil, thereby improving outcomes for individuals who use substances and their families.

## 2. Materials and Methods

We conducted a secondary analysis of data collected from a cross-sectional study on the characteristics of adult family members (age > 18 years old) affected by a relative who uses substances (RSU) in Brazil. A convenience sample of 3056 affected family members (AFM) was interviewed face-to-face by trained researchers between June 2012 and July 2013 to determine the sociodemographic, family, and psychological characteristics of AFM [31]. To our knowledge, this is the largest sample of AFM obtained through primary data collection to date. The nationwide scope of this study yielded a diverse sample, capturing regional variation across Brazil, a wide range of demographic and background characteristics, and differing reports regarding the relative’s primary substance and duration of substance-related problems, as well as varied family relationships between respondents and their affected relatives. Of the 3056 participants, 3030 responded to a question on whether they had sought help for the relative and were, thus, included in this analysis.

### 2.1. Procedure

This study received ethical approval from the Ethics Committee of the Faculty of Medicine at the Federal University of Sao Paulo (CEP 0499/2017, approval number 2.109.016). A questionnaire pack containing demographic and background questions about the AFM participant and their RSU was administered. The research team tracked down services that provided either support or treatment for substance use (therapeutic communities, rehabilitation clinics, Narcotics Anonymous, Alcoholics Anonymous) and self-help groups for AFM (‘Amor Exigente’, a networking group spread across the country that offers psychological support to families that have an RSU, and the group ‘Sobriedade’, a pastoral care movement from the Catholic Church in Brazil focused on the social problems of exclusion, poverty, and violence related to substance use) across the largest cities in each of the five Brazilian regions (North, Northeast, Central-West, Southeast, and South). A convenience sample of AFM participants was recruited. Participants recruited from residential/rehabilitation clinics were initially approached by the researchers in the waiting room of the service during visits to the RSU. In self-help groups, researchers orally invited and distributed a participant information sheet to family members during group sessions. There were no restrictions on participant characteristics (e.g., sex, age, relationship with the RSU). Questionnaires were administered in person by trained interviewers, taking an average of 40 minutes to complete. Interviews in residential/rehabilitation clinics were scheduled during family visiting times and group sessions for self-help groups. No payment was offered in Brazil, following local practices.

### 2.2. Measures

#### 2.2.1. Demographics

Participants’ age group, relationship status (married/long stable relationship/partner; yes/no answer), ethnic background (White versus other = Black/Mixed Ethnicity/Indigenous/other), and relation to the RSU (e.g., spouse, partner, child, sibling) were collected. Regarding socioeconomic status (SES), participants were also asked to report their educational level (no schooling/primary school only/secondary school only/higher education/postgraduate diploma), car ownership, and the number of bathrooms in the house. The inclusion of housemaid employment reflects a common indicator of higher income in Brazilian households, where the use of full-time domestic help is prevalent among affluent families. Given that educational level was significantly intercorrelated with car ownership, the number of bathrooms, and housemaids (0.29–0.53), a single SES variable was created by summing each of the four variables. This resulted in a variable with a range of 0–8 (Cronbach’s alpha of 0.71).

#### 2.2.2. Help-Seeking

Participants were asked if they had ever sought help for their relative and, if so, how many months it took for them to seek help since they discovered the substance use problem. Immediate help-seeking was defined as having sought help within 3 months after discovering the problem and was recorded as a binary variable (delay versus immediate help-seeking). Given the lack of evidence on the length of time for seeking help in AFM, the decision for this cut-off point was based on practical considerations, such as aligning with prior research on help-seeking behaviour for mental health and substance use issues, which often identifies early intervention within a few months as critical for better outcomes [32]. The variable length of time for seeking help was recorded into an ordinal variable ranging from 1 (from four months to one year) to 2 (from one to two years), 3 (from two to three years), 4 (from three to four years), and 5 (four years or more). Reasons for not seeking initial help for their relatives (yes/no answer) were also asked (i.e., not knowing where to seek help, thoughts about resolving the problem independently of help, a desire to hide the problem, financial difficulties, fear of the relative’s threats, and the relative not accepting help). This list of reasons was informed by prior research conducted in São Paulo, Brazil, which highlighted the interplay of informational, emotional, and structural barriers that often delay AFM’s engagement with support services [27]. Participants were also asked to report the first place they sought help for their relative including church/religious centres, mental health professionals (psychiatric/psychologists), general doctors, self-help groups (Narcotics Anonymous/Alcoholic Anonymous), community-based drug treatment services (Psycho-Social Care Centres for Alcohol and Drugs [CAPS-AD]), hospitals (general/residential hospitals), and social/justice services (social services/youth offending teams).

#### 2.2.3. Coping Strategies

The Brazilian-adapted version of the Coping questionnaire (COPE) [33] was administrated. The COPE was originally developed by Orford and colleagues [34] and was designed to obtain information about how the family members have coped with their relatives’ substance use in the last 3 months. Three main coping strategies are assessed: engaged (assertively addressing substance use or trying to control it), tolerant-inactive (sacrificing personal interests or being too fearful to act), and withdrawal (prioritising one’s own or family member’s interests first or avoiding the substance user). The Brazilian-adapted version comprises 24 items. Each item is scored on a Likert Scale (0 = never, 1 = rarely, 2 = sometimes, 3 = often). Average scores were calculated for the three subscales separately. Cronbach alphas for the engage, tolerant-inactive, and withdrawal coping subscales were 0.87, 0.75, and 0.62, respectively.

#### 2.2.4. Relative with Substance Use Problems

Participants were asked to report characteristics of the relative including gender, age, and substance of preference. The list of substances included the relative use of both licit (e.g., alcohol, sleeping pills, painkillers) and illicit (e.g., hallucinogens, amphetamines, ecstasy) substances, regardless of severity, pattern of use, and clinical diagnosis of a substance use disorder. Given that the most commonly used substances in Brazil are cannabis, cocaine, crack-cocaine, and alcohol [35,36,37], our analysis focused on these substances. Additionally, participants were asked about the length of time (in years) they had known about the substance use problem.

## 3. Analysis

Descriptive information for continuous variables was reported as mean and SD and for categorical variables as median and interquartile range (IQR). Categorical variables were reported as the count of non-missing observations and the percentage. To determine the association between potential risk factors for delayed support seeking for the relative, logistic regression models were run with immediate help seeking (yes/no) as the outcome regressed on sociodemographic and other characteristics. Separate models were run for each predictor, first unadjusted for potential confounders and then adjusted for potential confounders. The potential confounders selected were gender, age, ethnic group, level of socioeconomic status, and region of residence.

Further analysis was conducted to explore the differences in the length of time for seeking help. Ordinal logistic regression models were run for the subsample of those who had not sought immediate help, where the outcome was the length of time to seek help. Separate models were run for each predictor. Similar to the previous approach, unadjusted and adjusted models for potential confounders (gender, age, ethnic group, level of socioeconomic status, and geographic region of residence) were conducted.

The analysis plan was not pre-registered, and results should be considered as exploratory.

## 4. Results

### 4.1. Sample Characteristics

Detailed characteristics of the sample are presented in Table 1. Briefly, the age group median was 45 to 54 years old (IQR 35–65). Most participants were female (80.1%), from a White ethnic background (68.5%), had a partner (58.3%), and were parents of an RSU (61.1%). The median for socio-economic status was below the medium level of 4 (median 3; IQR 1–4) suggesting that the sample was characterised by participants from the lower end of the SES indicator used in this study. A greater proportion of participants were from the Southeast region of Brazil (42.0%) than the other regions. Most participants were related to a male RSU (94.1%) and had another relative in the family who also used substances (62.3%). The age mean for the RSU was 31.9 (SD 11.15). On average, participants reported lower scores on tolerance (mean 1.65, SD 0.63) coping strategies than engaged (mean 2.16; SD 0.85) and withdraw (mean 2.48; SD 0.99) coping strategies.

### 4.2. Help-Seeking Behaviours and Coping Strategies

As reported in Table 1, most participants had sought help for the relative (*N* = 2901, 92.7%). Compared to those who did not seek help, those who did were statistically significantly more likely to be from a higher socioeconomic group (OR 1.22, 95% CI 1.1; 1.4), to be from the Southeast region (OR 1.6, 95% CI 1.2; 2.2), and to have a relative who used cocaine (OR 2.3, 95% CI 1.4; 4.0). The likelihood of seeking help decreased for those participants from the Northeast region (OR 0.6, 95% CI 0.4; 0.8) and whose relatives had a problem with alcohol (CI 0.6, 95% CI 0.4; 0.8).

Two-thirds of the participants delayed seeking help for the relative after discovering the substance use problem (*N* = 1915; 66.0%). On average, it took 37.16 (SD 70.71) months (approximately 3 years) for the AFM to seek this help. The main reasons for delaying seeking help included the relative not accepting help (31.5%) and participants thinking that help/treatment was not needed (24.0%), along with a lack of knowledge about where to access care (20.6%). A variety of services were initially sought, with hospitals being reported by almost one quarter of the sample (23.0%) and self-help groups by 12% of the sample.

### 4.3. Factors Associated with Immediate Help-Seeking Behaviours

Table 2 describes the differences between AFM who sought help for their relatives soon after discovering the substance use problem and those who delayed seeking it. Compared to AFM who did not seek immediate help, those who did were statistically significantly more likely to be older (OR 1.1, 95% CI 1.0; 1.2), from a White background (OR 1.7, 95% CI 1.4; 2.0), from a higher SES (OR 1.3, 95% CI 1.2; 1.4), a resident in the Southeast region (OR 1.3, 95% CI 1.0; 1.5), the parent of the RSU (OR 1.7, 95% CI 1.4; 2.0), and have reported cannabis as the main substance of use of the relative (OR 1.4, 95% CI 1.2; 1.6). The odds of immediate help seeking statistically significantly decreased for those who are residents in the Northeast (OR 0.7, 95% CI 0.6; 0.9), who are siblings (OR 0.5, 95% CI 0.4; 0.6), whose relatives used alcohol (OR 0.4, 95% CI 0.3; 0.5), and who reported higher scores in the tolerant coping strategy (OR 0.9, 95% CI 0.8; 0.9). Among the first services accessed for help by AFM, mental health professionals and social/justice services statistically significantly increased the odds of immediate help-seeking behaviour (OR 2.6, 95% CI 2.1; 3.1), while attending hospitals was associated with a delay in help seeking (OR 0.6, 95% CI 0.5; 0.7). All these associations remained statistically significant after controlling for possible socioeconomic confounders.

### 4.4. Factors Associated with Length of Time for Seeking Help

**Figure 1:**
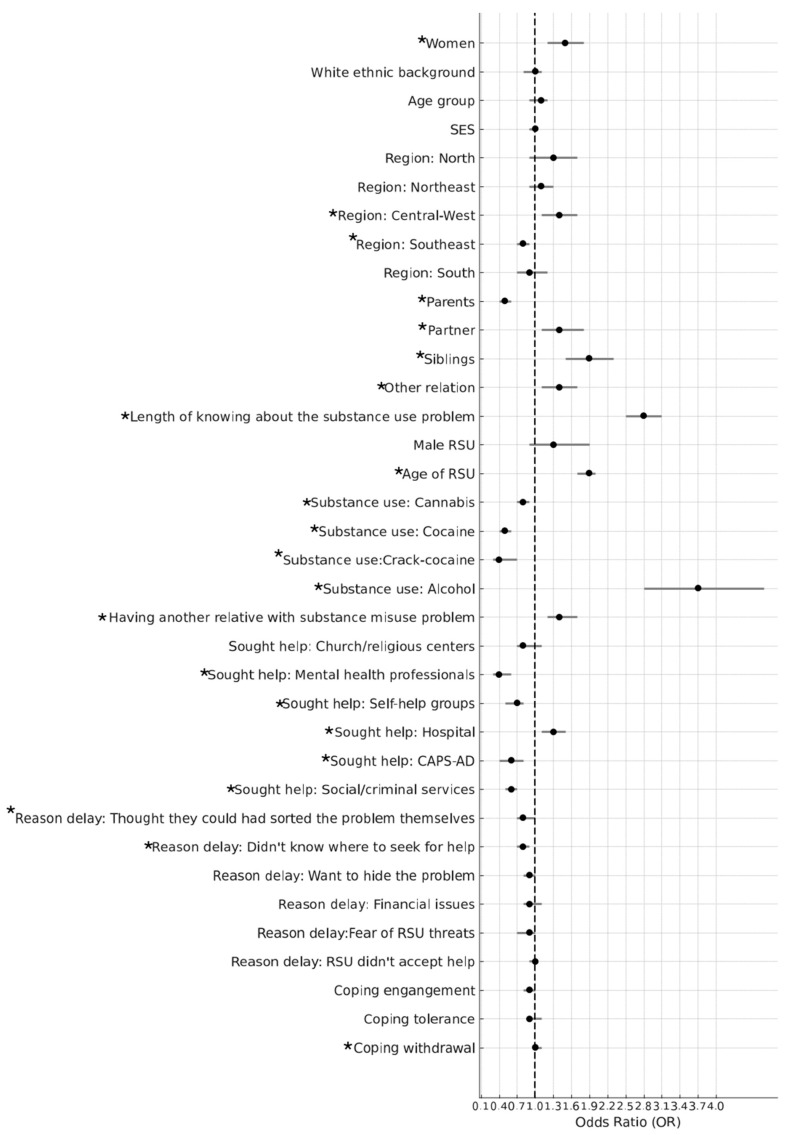
The associations between demographic factors, help-seeking behaviours, coping strategies, characteristics of the RSU, and length of time for those AFMs who delayed seeking help for their relative (*N* = 1915). Note: The bars represent 95% CIs for the difference between the length of time for a 1-unit change in the predictor variable for continuous variables and change relative to the reference group (e.g., ‘men’ vs. ‘women’) for categorical variables (adjusted models). The factors in the figure which can be seen to have a significant association with the length of time are marked with a star (*) for clarity.

A longer length of time for seeking help for the relative was statistically significantly associated with the following variables: female AFM (OR 1.5, 95% CI 1.2; 1.8), residents in the Central-West region (OR 1.4, 95% CI 1.1; 1.7), a longer length of time knowing about the substance use problem (OR 2.8, 95% CI 2.5; 3.1), an older RSU (OR1.9, 95% CI 1.7; 2.0), having another relative in the family who uses substances (OR 1.4, 95% CI 1.2; 1.7), and with high levels of withdrawal coping strategy (OR 1.10, 95% CI 1.1; 1.2). A longer length of time was also statistically significantly associated with seeking initial support in hospitals (OR 1.3, 95% CI 1.1; 1.5). Participants who sought initial help from mental health professionals (OR 0.4, 95% CI 0.3; 0.7), in self-help groups (OR 0.7, 95% CI 0.5; 0.9), in CAPS-AD (OR 0.6, 95% CI 0.4; 0.8), and with social/criminal services (OR 0.6, 95% CI 0.5; 0.7) were statistically significantly less likely to delay the length of time for seeking help. Participants from the Southeast region sought help in a statistically significantly shorter length of time compared to those AFM from the other geographic regions (OR 0.8, 95% CI 0.7; 0.9). Parents also sought help in a significantly shorter length of time than partners, siblings, and other relatives (OR 0.5, 95% CI 0.4; 0.6). The use of illicit drugs (cannabis, cocaine, and crack cocaine) was also statistically significantly associated with a shorter length of time for seeking help, while the use of alcohol was associated with a longer length of time (OR 3.7, 95% CI 2.8; 4.8). Of all the reasons for delaying help-seeking, not knowing where to go for help was the only reason statistically associated with the length of time for seeking help (OR 0.8, 95% CI 0.7; 0.9). All these associations remained statistically significant after controlling for possible socioeconomic confounders. Figure 1 illustrates the associations between factors and the length of time for seeking help. Odds ratios for adjusted and unadjusted models are reported in the Supplementary File (Table S1).

## 5. Discussion

This cross-sectional study investigated help-seeking behaviours by AFM in Brazil. The findings suggest that most AFM sought help for their relatives; however, two-thirds of the participants delayed seeking this help. Determining whether this figure is high or low requires careful consideration because little comparable data exist relating to the prevalence of AFMs’ help-seeking behaviours for their RSU. Nevertheless, we found that, on average, it took approximately 3 years for AFM to seek help for their relative, which is the same average length of time taken by AFMs in Brazil to seek help for themselves [27].

Our findings also reveal disparities in support seeking across socioeconomic groups, geographic regions in Brazil, and the type of substance used by the relative. AFM from lower socioeconomic groups, those from the Northeast region, and those whose relatives had a problem with alcohol use were less likely to seek help. Yet, AFM from the Southeast region and those whose relatives use cocaine were more likely to seek support. Immediate help-seeking was associated with AFM from higher socioeconomic groups and those from the Southeast region. White AFM were also more likely to seek immediate help than non-Whites, further suggesting that barriers to seeking help might be rooted in social disadvantages [38]. Like other large low-middle-income countries, these disparities reflect regional inequalities in social and health services, with the inequitable distribution of skilled staff hindering healthcare delivery, especially in poorer regions like the Northeast and North [39,40,41,42]. While the federal government allocates funds for public health, states and municipalities are responsible for distributing healthcare resources locally. São Paulo, one of Brazil’s wealthiest states in the Southeast, receives more funding and invests heavily in healthcare, benefiting from a higher concentration of healthcare professionals due to the presence of prestigious medical universities and the state’s appeal to professionals from across Brazil and abroad. Critical to improving the engagement of AFM with health services is addressing regional inequalities, enhancing access to resources, and overcoming social barriers. This includes increasing the availability of skilled healthcare professionals, particularly in underserved areas, and ensuring the equitable distribution of healthcare funding and services across regions. National initiatives to train and distribute healthcare and social workers more effectively, alongside targeted interventions to educate AFM, especially in lower socioeconomic and marginalised groups, are essential to improving service engagement and support for families affected by substance use.

The fact that hospitals were the most sought-after place for help highlights that seeking help directly from a public health service is an important route for AFM. However, given that hospitals as the first place of help were associated with delay and a longer length of time to seek help, it might be that families reaching these places do it during a time of crisis (e.g., physical health deterioration/injuries due to substance use, drug overdose). The findings also show that other formal resources are being sought out by AFMs. Those who sought immediate help were more likely to go to social/justice services. A shorter length of time for seeking help was also associated with social/justice services as well as CAPS-AD (community-based drug treatment services) and mental health professionals. These findings highlight the need for formal resources including social/criminal and healthcare settings to be equipped to respond to the request for help coming from AFM. This could involve training professionals in these services to have a nonjudgemental attitude toward family members, as professionals in such services often harbour biases against families, such as having a role in causing or sustaining substance use, and lack skills or motivation to pursue family involvement in the substance use treatment [43,44].

Concerning the type of substance use by the relative, alcohol was the only substance associated with a longer delay in help-seeking by AFM. This finding aligns with previous research indicating that alcohol-related problems are often perceived as less severe or more socially acceptable compared to illicit drug use, which may contribute to delays in recognising the need for external support [5]. Misconceptions surrounding alcohol dependence such as the belief that it can be controlled without professional help or that it is a moral failing rather than a health condition [45] can further discourage timely help-seeking. Our findings highlight the need for improving community-level education and information resources to challenge stigma and misinformation. In particular, integrating substance use education into primary care services could facilitate earlier identification and support for families affected by problematic alcohol use [46].

In line with other studies, the most reported barriers to help-seeking behaviours include beliefs that help/treatment is not needed and a lack of knowledge about where to access care [47,48]. This is possibly due to both self-stigma and a lack of resources to learn more about viable treatment options [44,49]. It is also important to note that many AFM never sought help for their relative. A potential strategy for improving AFMs’ willingness to seek help for their relative is by increasing family awareness of substance-related problems and treatment through public health strategies directly targeting families (e.g., websites, brochures displayed in primary care offices and schools). However, it does not mean that, when AFM access services for help, they are advised on all possible sources of help and the process of care planning is initiated. Given the lack of guidelines in Brazil, many AFM might experience difficulties negotiating the complex service network they must navigate to help their relative. We suggest establishing a national framework to enhance coordination among social and health services and promote integrated approaches to improve substance use treatment services and family support services. The successful implementation of this framework has the potential to enhance awareness among family members regarding available support for both them and other service providers.

Lastly, we found that higher scores in the tolerant-inactive coping strategy were associated with not seeking immediate help, while the withdrawal coping strategy was associated with a longer length of time to seek help. According to the stress–strain–coping-support model [50], AFM adopting tolerant-inactive coping tend to accept or support substance use and promote self-sacrificing behaviour on the part of the relative. The withdrawal coping strategy refers to techniques that encourage family members to focus on their own needs and to increase the distance between them and their RSU. There is some evidence suggesting that tolerant-inactive and withdrawal coping styles might lead to more negative outcomes for AFM [5]. Evidence also shows that families are powerful resources for enhancing treatment and recovery success among individuals who use substances [13,14,15]. Yet, in Brazil, families are not routinely targeted or systematically included in clinical practice. Our findings emphasise the importance of research-based family engagement interventions across substance use treatment services. Brazilian AFM might benefit from support provided by the Five-Step Method [51] and/or the Motivational Intervention for Families (MIF) [52], which are interventions designed to help AFM cope with distress and develop communication skills and problem solving. As noted by Rane and colleagues [53], in low- and middle-income countries like Brazil, where limited resources and competing health and social priorities present significant challenges, further research is needed to adapt family interventions developed and tested in higher-income countries. Given the collective cultural nature of these countries, priority should be given to group-based interventions and delivery strategies that leverage lay health workers, ensuring a wider reach and greater impact in supporting affected families.

### Strengths and Limitations

A key strength of this study is the large and representative characteristics of the sample of AFM including the type of relationships, substances used by the relative, and regional differences within a country. We do, however, recognise that the period when the data were collected (2012–2013) might raise concerns about its relevance in the present context, particularly given the societal and healthcare shifts that have occurred since the COVID-19 pandemic that might have impacted the way people seek help. Nevertheless, it is important to highlight that no national efforts have been made in Brazil to encourage the inclusion of the family in substance use treatment, and little has changed in the provision of substance use treatment across the country in the past decade [54]. As a result, substance use treatment services remain highly fragmented, with limited coordination among healthcare providers and social services, creating additional challenges for Brazilians to access effective and timely support.

We recognise that this study is limited by its cross-sectional design and that the nature of these questions allows only for an introductory understanding of help-seeking behaviour for RSU from Brazilian AFM. Moreover, help-seeking behaviour might be underreported by the participants due to recall bias, a type of systematic error that occurs when individuals do not accurately remember or report past events. In this context, participants may forget or misrepresent previous efforts to seek help, leading to an underestimation of actual help-seeking behaviour. Given that participants were recruited from substance use self-help groups and treatment services, this convenience sampling approach might have introduced sampling bias and might have also influenced some of our findings. For example, the data variance could be limited due to similar characteristics of those family members who have been receiving some form of information/support regarding the relative’s substance use. It is also important to recognise that, while the survey question was about the AFM seeking help for their relatives and not about whether the RSU was receiving support, we acknowledge that recruiting AFM via substance use treatment/support services might have overestimated the prevalence of participants who sought support. Moreover, family members were included in the study based on their engagement with services related to substance use, regardless of whether their relative had a formal clinical diagnosis of substance use disorder. Although the findings indicate that, on average, the relative’s substance use problem had been ongoing for a substantial period (mean = 8.9 years, SD = 8.6), suggesting a prolonged and embedded issue within the family, data on the specific patterns and severity of the relative’s substance use were not collected. This limits the depth of our analysis and our ability to fully contextualise the help-seeking behaviours reported. Attention is required when interpreting findings from the Brazilian-adapted version of the Coping questionnaire (COPE), particularly regarding the low reliability of the withdrawal subscale [33]. This low reliability raises concerns about whether the data accurately reflect the intended concept, potentially leading to misleading or erroneous interpretations. The careful evaluation of the subscale’s items and their alignment with the construct being measured is essential to ensure valid conclusions.

## 6. Conclusions

While most of the AFMs sought help for their relatives, only one-quarter did so soon after discovering the problem. A range of services was sought as the first place for help, with preferences tending towards formal resources, such as hospitals, health professionals, or social/criminal services. Our findings highlight significant disparities in help-seeking behaviour across socioeconomic groups, geographic regions in Brazil, and the type of substance used by the relative. These disparities point to the critical need for national initiatives to enhance the training and equitable distribution of healthcare and social workers, particularly in underserved regions. Moreover, targeted interventions are essential to educate AFMs on the importance of timely engagement with healthcare services, which can lead to improved outcomes for both families and their relatives. The factors associated with help-seeking behaviours identified in this study should guide the development/adaptation of evidence-based family engagement interventions tailored to the Brazilian context.

## Figures and Tables

**Table 1:** Characteristics of the total sample according to whether the participant had sought help or not for the relative with substance use problems.

	Total Sample (*N* = 3030)	Did Not Seek Help (*N* = 229; 8.3%)	Sought Help (*N* = 2901; 92.7%)	OR (95% CI) ^1^
Women	2493 (80.1%)	185 (81.1%)	2308 (80.0%)	0.93 (0.88, 1.31)
White ethnic background	2145 (68.5%)	144 (62.3%)	2001 (69.0%)	1.31 (0.99, 1.73)
Age group				1.07 (0.94, 1.23)
18 to 24	118 (3.8%)	16 (7.0%)	102 (3.5%)	
25 to 34	330 (10.5%)	19 (0.8.3%)	311 (10.7%)	
35 to 44	510 (16.3%)	38 (16.6%)	442 (16.3%)	
45 to 54	959 (30.6%)	74 (32.1%)	885 (30.5%)	
55 to 64	820 (26.2%)	54 (23.5%)	766 (26.4%)	
65 and over	393 (12.6%)	28 (12.3%)	365 (12.6%)	
Married/long stable relationship/partner	1826 (58.3%)	158 (57.4%)	1673 (58.0%)	1.42 (0.98; 2.04)
SES (0 lower to 8 higher)(median; quartile)	3 (1; 4)	2 (1; 4)	3 (2; 5)	**1.22 (1.06, 1.41)**
Region of residence				
North	277 (8.8%)	22 (9.6%)	255 (8.8%)	0.91 (0.57, 1.43)
Northeast	787 (25.1%)	81 (35.4%)	706 (24.3%)	**0.59 (0.44, 0.78)**
Central-West	372 (11.9%)	28 (12.2%)	344 (11.9%)	0.96 (0.64, 1.46)
Southeast	1314 (42%)	72 (31.4%)	1242 (42.8%)	**1.63 (1.22, 2.18)**
South	380 (12.1%)	26 (11.3%)	354 (12.2%)	1.08 (0.71, 1.66)
Relation to the RSU				
Parents	1912 (61.1%)	132 (57.6%)	1789 (61.4%)	1.17 (0.89, 1.53)
Partner	429 (13.7%)	24 (10.5%)	405 (14.0%)	1.38 (0.90, 2.14)
Siblings	394 (12.6%)	35 (15.3%)	359 (12.4%)	0.78, 0.53, 1.14)
Other (child/nephew/grandparents/in-laws)	395 (12.6%)	38 (16.6%)	357 (12.3%)	0.70 (0.49, 1.02)
Length of time knowing about the substance use problem (years) (Mean, SD)	8.9 (8.6)	9.4 (9.3)	8.9 (8.5)	0.94 (0.83, 1.03)
Male, RSU	2947 (94.1%)	214 (93.4%)	2733 (94.2%)	1.14 (0.66, 1.96)
Age of the relative (Mean, SD)	31.9 (11.15)	31.9 (11.1)	31.7 (12.2)	1.02 (0.89, 1.16)
Substance of problem				
Cannabis	2141 (68.4%)	155 (68.7%)	1986 (68.5%)	1.04 (0.78, 1.38)
Cocaine	425 (13.6%)	15 (6.5%)	410 (14.1%)	**2.35 (1.38, 4.00)**
Crack-cocaine	105 (3.3%)	9 (3.9%)	96 (3.3%)	0.84 (0.42; 1.67)
Alcohol	366 (11.7%)	42 (18.3%)	324 (11.2%)	**0.56 (0.39, 0.78)**
Having more than one relative with substance use problem	1929 (62.3%)	149 (66.8%)	1780 (61.9%)	0.80 (0.60, 1.07)
Coping strategies (Mean, SD)				
Engaged	2.16 (0.84)	2.20 (0.86)	2.16 (0.84)	0.94 (0.83, 1.08)
Tolerant-inactive	1.65 (0.63)	1.7 (0.64)	1.6 (0.62)	0.92 (0.81, 1.05)
Withdrawal	2.48 (0.99)	2.6 (1.02)	2.5 (1.0)	0.88 (0.77, 1.01)
First place where help was sought *		-	-	
Church/Religion centres	328 (11.1%)	-	-	
Mental health professionals	509 (16.3%)	-	-	
General doctors	50 (1.6%)	-	-	
Self-help groups	394 (12.6%)	-	-	
Hospitals	721 (23.0%)	-	-	
CAPS-AD	212 (6.8%)	-	-	
Social/Criminal services	648 (20.7%)	-	-	
Reasons for not seeking initial help for the relative		-	-	
Thought they could sort the problem by themselves	499 (24.0%)	-	-	
Did not know where to seek for help	429 (20.6%)	-	-	
Wanted to hide the problem	99 (4.8%)	-	-	
Financial issues	40 (1.9%)	-	-	
Fear of the RSU’s threats	21 (1.0%)	-	-	
RSU did not accept	656 (31.5%)	-	-	

Note: RSU = relative with substance use problems; ^1^ unadjusted ORs; * mental health professionals (psychiatric/psychologists), self-help groups (Narcotics Anonymous/Alcoholic Anonymous), CAPS-AD (community-based drug treatment services), hospitals (general/residential hospitals), and social/justice services (social services/youth offending teams). In bold: *p* < 0.05.

**Table 2:** Factors associated with immediate help seeking for the relative by the AFM (*N* = 2901).

	Delayed (*N* = 1915)	Immediate (*N* = 986)	Unadjusted OR (95% CI)	Adjusted OR (95% CI) ^a^
Women	1669 (80.7%)	824 (78.7%)	0.88 (0.73; 1.05)	1.01 (0.83; 1.21)
White ethnic background	1351 (65.0%)	794 (75.5%)	**1.66 (1.41; 2.00)**	**1.32 (1.11; 1.59)**
Age group (median, quintiles)	4 (3; 5)	4 (3; 5)	**1.14 (1.1; 1.23)**	**1.11 (1.03; 1.20)**
SES (median, quintiles)	2 (1; 4)	3 (2; 5)	**1.34 (1.24; 1.44)**	**1.28 (1.18; 1.38)**
Region of residence				
North	197 (9.5%)	80 (7.6%)	0.79 (0.60; 1.03)	0.90 (0.68; 1.19)
Northeast	563 (27.1%)	224 (21.3%)	**0.73 (0.61; 0.87)**	**0.77 (0.64; 0.92)**
Central-West	247 (11.9%)	125 (11.9%)	1.01 (0.80; 1.26)	0.97 (0.78; 1.22)
Southeast	830 (39.9%)	484 (46.0%)	**1.28 (1.10; 1.49)**	**1.20 (1.03; 1.40)**
South	242 (11.6%)	138 (13.1%)	1.14 (0.92; 1.43)	1.13 (0.89; 1.42)
Relation to the RSU				
Parents	728 (38.1%)	1184 (61.9%)	**1.70 (1.45; 1.99)**	**1.71 (1.42; 2.06)**
Partner	312 (72.7%)	117 (27.3%)	**0.71 (0.56; 89)**	0.79 (0.62; 1.02)
Siblings	313 (79.4%)	81 (20.6%)	**0.47 (0.36; 61)**	**0.48 (0.37; 0.63)**
Other (child/nephew/grandparents/in-laws)	270 (13.0%)	125 (11.9%)	0.90 (0.72; 1.13)	0.98 (0.77; 1.23)
Length of time knowing about the substance misuse problem (years)	9.8 (8.9)	7.1 (7.7%)	**0.70 (0.64; 0.76)**	**0.95 (0.94; 0.96**)
Male, RSU	1965 (94.5%)	982 (93.0%)	0.82 (0.61; 1.12)	0.86 (0.63; 1.18)
Age of the relative (Mean, SD)	32.9 (11.4)	29.9 (10.2)	0.75 (0.70; 0.82) *	**0.73 (0.67; 0.80)**
Substance of problem				
Cannabis	1373 (66.0%)	768 (73.0%)	**1.39 (1.18; 1.64)**	**1.32 (1.12; 1.56)**
Cocaine	271 (13.0%)	154 (14.6%)	1.14 (0.92; 1.41)	1.14 (0.91; 1.41)
Crack-cocaine	66 (3.2%)	39 (3.7%)	1.17 (0.78; 1.76)	1.33 (0.88; 2.02)
Alcohol	303 (14.6%)	63 (6.0%)	**0.37 (0.28, 49)**	**0.40 (0.30; 0.53)**
Have more than one relative with substance use problems	1322 (64.1%)	607 (58.4%)	**0.78 (0.67; 0.91)**	**0.81 (0.60; 0.94)**
First place where help was sought *				
Church/Religion centres	239 (11.5%)	109 (10.4%)	0.89 (0.70; 1.13)	0.95 (0.74; 1.21)
Mental Health clinical professionals	243 (11.7%)	266 (25.3%)	**2.56 (2.11; 3.10)**	**2.16 (1.78; 2.64)**
General doctors	30 (1.4%)	20 (1.9%)	1.32 (0.75; 2.34)	1.34 (0.75; 2.41)
Self-help groups	247 (11.9%)	147 (14.0%)	1.21 (0.97; 1.50)	1.11 (0.89; 1.39)
Hospital	535 (25.7%)	186 (17.7%)	**0.62 (0.51; 0.75)**	**0.68 (0.56; 0.82)**
CAPS-AD	128 (6.2%)	84 (8.0%)	1.32 (0.99; 1.76)	1.35 (0.99; 1.80)
Social/Criminal services	409 (19.7%)	239 (22.7%)	**1.20 (1.01; 1.43)**	**1.29 (1.07; 1.55)**
Coping strategies (mean, SD)				
Engaged	2.16 (0.85)	2.17 (0.83)	1.01 (0.94; 1.09)	1.03 (0.94; 1.13)
Tolerant-inactive	2.07 (0.64)	1.60 (0.60)	**0.88 (0.81; 0.94)**	**0.85 (0.75; 0.96)**
Withdrawal	2.48 (1.01)	2.46 (0.99)	0.98 (0.91; 1.06)	0.92 (0.85; 1.01)

Note: Immediate help-seeking defined as having sought help within 3 months after discovering the problem. RSU = relative with substance use problems; * mental health professionals (psychiatric/psychologists), self-help groups (Narcotics Anonymous/Alcoholic Anonymous), CAPS-AD (community-based drug treatment services), hospitals (general/residential hospitals), and social/justice services (social services/youth offending teams). ^a^ Adjusted ORs for gender, age, ethnic group, level of SES, and geographic region of residence. In bold: *p* < 0.05.

## Data Availability

The data presented in this study are available on request from the corresponding author due to ethical reasons.

## Supplementary Materials

The following supporting information can be downloaded at: https://www.mdpi.com/article/10.3390/ijerph22060968/s1, Table S1. Factors associated with the length of time for seeking help for the relative.
