# Supplementary material for: Family Members’ Help-Seeking Behaviour for Their Relative Who Uses Substances: A Cross-Sectional National Study in Brazil
Source: Int J Environ Res Public Health. 2025 Jun 19;22(6):968. doi: 10.3390/ijerph22060968 (PMC12192736; doi:10.3390/ijerph22060968)
Supplement: Supplementary file 1 [file ijerph-22-00968-s001.zip › ijerph-3635848-supplementary.pdf]

Table S1 Factors associated with the length of time for seeking help for the relative

	OR (95%CI) Unadjusted	OR (95%CI) Adjusted
Women	**1.5 (1.2; 1.8)**	**1.4 (1.2; 1.8)**
White ethnic background	1.0 (.8; 1.1)	1.0 (0.8; 1.2)
Age group	1.1 (.9; 1.2)	1.1 (0.9; 1.2)
SES (0 lower to 8 higher)	1.0 (.9; 1.0)	1.0 (0.9; 1.0)
Region of residence		
North	1.3 (0.9; 1.7)	1.2 (0.9; 1.6)
Northeast	1.1 (0.9; 1.3)	1.0 (0.9; 1.2)
Central-West	**1.4 (1.1; 1.7)**	**1.3 (1.0; 1.7)**
Southeast	**0.8 (0.7; 0.9)**	**0.8 (0.7; 0.9)**
South	0.9 (0.7; 1.2)	0.9 (0.7; 1.2)
Relation to the RSU		
Parents	**0.5 (0.4; 0.6)**	**0.3 (0.3; 0.4)**
Partner	**1.4 (1.1; 1.8)**	**1.5 (1.1; 1.9)**
Siblings	**1.9 (1.5; 2.3)**	**2.2 (1.7; 2.8)**
Other	**1.4 (1.1; 1.7)**	**1.5 (1.2; 2.0)**
Length of time knowing about the substance misuse	**2.8 (2.5; 3.1)**	**2.9 (2.6; 3.2)**
Male, RSU	1.3 (0.9; 1.9)	1.3 (0.9; 1.8)
Age of RSU	**1.9 (1.7; 2.0)**	**1.9 (1.7; 2.0)**
Substance of problem		
Cannabis	**0.8 (0.7; 0.9)**	**0.8 (0.7; 0.9)**
Cocaine	**0.5 (0.4; 0.6)**	**0.5 (0.4; 0.6)**
Crack-cocaine	**0.4 (0.3; 0.7)**	**0.4 (0.3; 0.7)**
Alcohol	**3.7 (2.8; 4.8)**	**3.6 (2.2; 4.0)**
Having another relative with substance misuse problem	**1.4 (1.2; 1.7)**	**1.4 (1.2; 1.6)**
First place that sought help for the relative *		
Church/Religion centres	0.8 (0.7; 1.1)	0.8 (.6; 1.1)
Mental Health clinical professionals	**0.4 (0.3; .56)**	**0.4 (0.2; 0.5)**
Self-help groups	**0.7 (0.5; .88)**	**0.7 (0.5; 0.9)**
Hospital	**1.3 (1.1; 1.5)**	**1.3 (1.0; 1.5)**
CAPS-AD	**0.6 (0.4; 0.8)**	**0.6 (0.4; 0.8)**
Social/Criminal services	**0.6 (0.5; 0.7)**	**0.6 (0.5; 0.7)**
Reasons for not seeking initial help for the relative		
Thought could sort the problem by themselves	0.8 (0.7; 1.0)	0.9 (0.7; 1.1)
Did not know where to seek for help	**0.8 (0.7; 0.9)**	**0.8 (0.7; 0.8)**
Want to hide the problem	0.9 (0.8; 1.0)	0.9 (0.8; 1.1)
Financial issues	0.9 (0.8; 1.1)	0.9 (0.8; 1.1)
Fear of the RSU' threats	0.9 (0.7; 1.0)	0.7 (0.7; 1.0)
RMS did not accept	1.0 (0.9; 1.0)	0.9 (0.9; 1.0)
Coping strategies		
Engagement	0.9 (0.8; 1.0)	0.9 (0.8; 1.0)
Tolerance	0.9 (0.9; 1.1)	0.9 (0.9; 1.3)
Withdrawal	**1.0 (1.0; 1.1)**	**1.10 (1.1; 1.2)**

Note: RSU = Relative with substance use problems; *mental health professionals (psychiatric/psychologists), self-help groups (Narcotics Anonymous/Alcoholic Anonymous), CAPS-AD (community-based drug treatment services), hospitals (general/residential hospitals) and social/justice services (social services/youth offending teams). [a] Adjusted ORs for gender, age, ethnic group, level of SES and geographic region of residence. In bold p<.05.
